# The distinct roles of mesenchymal stem cells in the initial and progressive stage of hepatocarcinoma

**DOI:** 10.1038/s41419-018-0366-7

**Published:** 2018-03-01

**Authors:** Chen Zong, Hangjie Zhang, Xue Yang, Lu Gao, Jing Hou, Fei Ye, Jinghua Jiang, Yang Yang, Rong Li, Zhipeng Han, Lixin Wei

**Affiliations:** 1 Tumor Immunology and Gene Therapy Center, Third Affiliated Hospital of Second Military Medical University, 200438 Shanghai, China; 2 Institute for Viral Disease Control and Prevention, Chinese Center for Disease Control and Prevention, Beijing, China; 3 Department of Pharmacy, Changhai Hospital, The Second Military Medical University, Shanghai, China

## Abstract

Increasing evidences suggest that mesenchymal stem cells (MSCs) could migrate to the tumor site and play a vital role in tumorigenesis and progression. However, it is still a lively debate whether MSCs exert a pro- or anticancer action. Cancer development and progression is a multistep process. Therefore, we investigated the effect of MSCs on hepatocarcinoma and whether the role of MSCs depends on the stage of cancer development. In our study, chronically exposing rats to *N*-diethylnitrosamine (DEN) was employed as hepatocarcinoma model. And to evaluate the effect of MSCs on hepatocarcinoma, the animals were divided into three groups: rats were injected with MSCs in the initial (DEN + MSC (Is) group) or progressive stage (DEN + MSC (Ps) group) of hepatocarcinoma, respectively. Rats injected with PBS were used as control (DEN group). Interestingly, we found that MSCs had a tumor-suppressive effect in the Is of hepatocarcinoma, yet a tumor-promotive effect in the Ps. In the Is, MSCs showed a protective role against drug damage, possibly through reducing DNA damage and ROS accumulation. Meanwhile, MSCs in the Is also exhibited anti-inflammatory and anti-liver fibrosis effect. Further, in the Ps, MSCs facilitated tumor formation not only by enhancing cancer cell proliferation but also through promoting stem cell-like properties and epithelial–mesenchymal transition of liver cancer cells. Taken together, MSCs have a paradoxical role in the different stages of hepatocarcinogenesis, which sheds new light on the role of MSCs in hepatocarcinoma and cautions the therapeutic application of MSCs for liver cancer.

## Introduction

In the past 50 years, much of our focus in cancer biology and therapy has been on genetic and epigenetic alterations in malignant cells^1^. However, it is now quite clear that tumor cells do not act alone. The cross-talk between tumor cells and surrounding stroma has gained increasing attention, due to their contribution to carcinogenesis, tumor invasion, and metastasis^2^. Among several cell types constituted the tumor stroma, the discovery about mesenchymal stem cells (MSCs) has achieved notoriety in recent years, for MSCs have a strong tropism into tumor where they are not only components, but also regulators^3,4^. A number of publications have highlighted an important effect of MSCs in tumor initiation, progressions, and metastasis^5–8^. The specific role and mechanism of MSCs in the development of cancer, however, remains unclear.

Hepatocellular carcinoma (HCC) is the third-leading cause of cancer mortality worldwide. However, the ineffective treatment and poor prognosis are common for lack of knowledge about mechanism of hepatocarcinogenesis^9^. Many literatures highlights that MSCs could migrate to sites of tissue injury, where they participate in wound repair and even as a key modulator of hepatocarcinogenesis^10,11^. However, whether MSCs suppress or promote HCC development is still contentious. Several studies indicated that MSCs restrain liver cancer growth by secreting cytokines or regulating cell cycle^12^, whereas others showed that MSCs promoted tumor progression and metastasis in animal models^13,14^. Therefore, making it clear how MSCs worked in HCC process will contribute to a deep understanding on hepatocarcinogenesis.

The transplantation tumor model was employed in most publications about MSCs and HCC, however which could not simulate how human primary tumor occurred. In our study, *N*-diethylnitrosamine (DEN) induced rat liver cancer model was used for providing a multistage hepatocarcinoma development resembling human primary HCC. To evaluate the effect of MSCs on HCC, rats were injected with MSCs in the initial (Is) or progressive (Ps) stage of hepatocarcinoma process, respectively. The results suggested that MSCs in the Is-prevented tumor growth by attenuating liver damage depending on reduced DNA damage, reactive oxygen species (ROS) accumulation, and inflammation. However, MSCs in the Ps facilitated tumor formation by enhancing stem cell properties and inducing EMT of liver tumor cell. These studies demonstrated that the timing of MSCs administration is the key point when MSCs are applied for live diseases, especially liver cancer treatment in the future.

## Results

### MSCs have a tumor-suppressive effect in the Is of hepatocarcinoma development and a tumor-promotive effect in the Ps

**Fig. 1:**
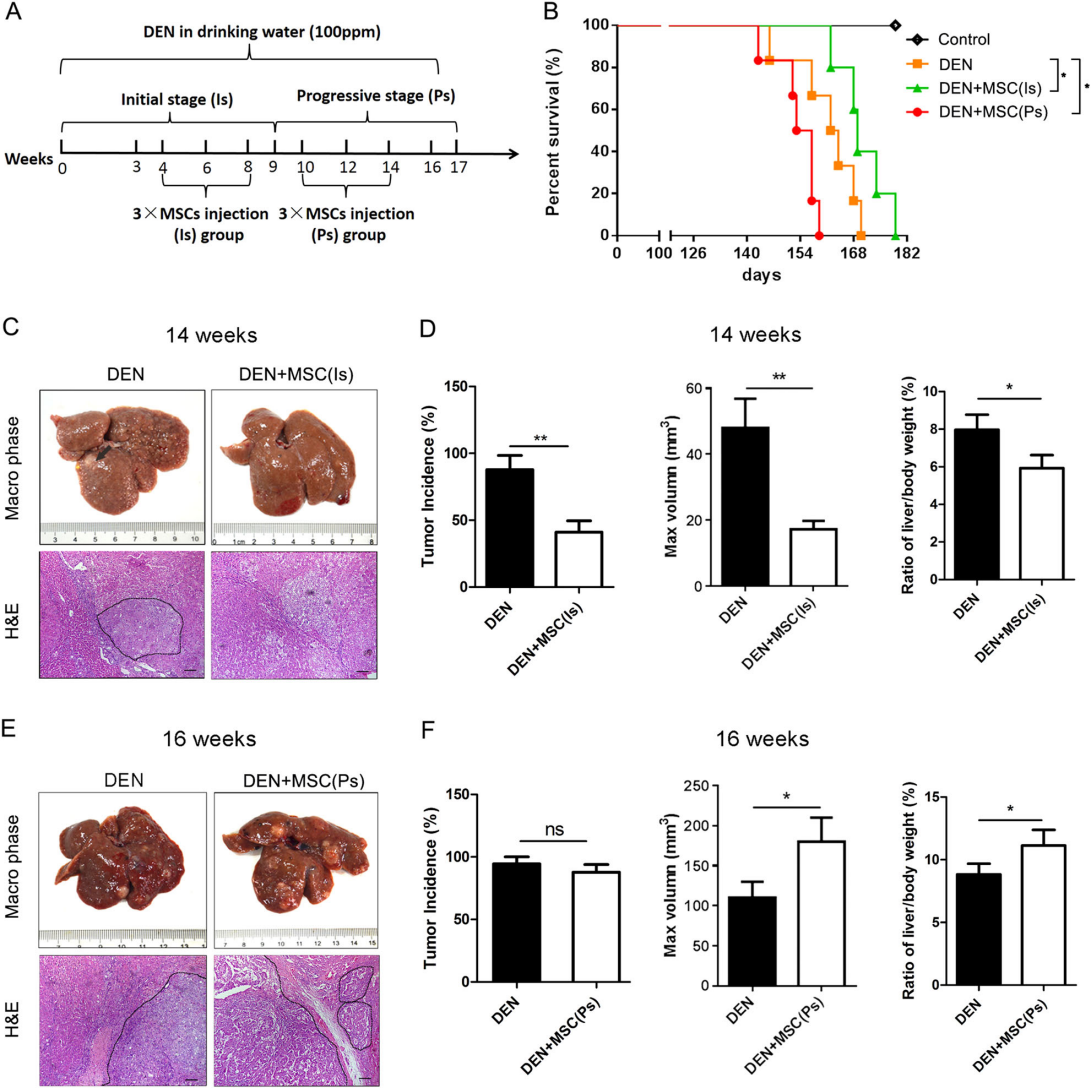
MSCs suppressed tumor formation in the Is of hepatocarcinoma development and promoted tumor development in the Ps. **a** A diagram of experimental protocol. Rats were administrated DEN (100 p.p.m., 95 mg/L) in drinking water for 14 weeks. DEN + MSC (Is) group was transplanted MSCs (1 × 10^6^) by intravenous injection at 4th, 6th, 8th week, and DEN + MSC (Ps) group was transplanted MSCs (1 × 10^6^) as the same method at the 10th, 12th, 14th week, respectively. **b** Survival cures of DEN, DEN + MSC (Is) and DEN + MSC (Ps) groups. **c**, **e** Gross and H&E staining appearance of livers in DEN and DEN + MSC (Is) group at 14 weeks **c** or DEN and DEN + MSC (Ps) group at 16 weeks after DEN treatment **e**. Dashed lines indicate the tumor area. **d**, **f** Tumor incidence, max tumor volume, and ratio of liver/body weight of DEN and DEN + MSC (Is) group at 14th week **d**, or DEN and DEN + MSC (Ps) group at 16th week **f**. Values are shown as mean ± SEM (*P < 0.05, **P < 0.01). Representative of three independent experiments. The area in the dotted line was shown as tumor area. ns no significant difference

Chronically exposing to diethylnitrosamine (DEN) provides a multistage hepatocarcinogenesis model. According to our previous study^15^, the hepatocarcinoma induced by DEN can be divided into two stage: the time from 0th to 9th week was regarded as the Is, and 10th to 17th week as the Ps. MSCs were injected by tail vein at the Is or Ps to explore the effect of MSCs in different stage of HCC development. Rats were divided into three groups: a. DEN; b. DEN + MSC (Is); c. DEN + MSC (Ps). In addition to DEN treatment, the rats in group DEN and DEN + MSC (Is) were injected with PBS or MSCs/MSCs-green fluorescent protein (GFP) every 2 weeks from the 4th to 8th week. Similarly, the DEN and DEN + MSC (Ps) was treated at the same frequency from the 10th to 14th week (Fig. 1a). Importantly, MSCs-GFP were used for tracing the MSCs in vivo, and we found GFP-positive MSCs in DEN + MSC (Is) and DEN + MSC (Ps) groups after DEN treatment, indicating that MSCs could migrate to site of liver tissue with damage (Supplementary Figure 1).

In a cohort of rats monitored for survival, the mean survival time of DEN + MSC (Is) group was better than that of DEN group at 14th week (Fig. 1b). Furthermore, the tumor incidence and the maximal tumor diameters decreased in DEN + MSC (Is) compared to DEN group (Fig. 1c, d). Interestingly, at 16th week, DEN + MSC (Ps) exhibited a shorter mean survival time compared with DEN group. And the maximal diameters of tumors and ratio of liver/body weight were significantly increased in DEN + MSC (Ps) group (Fig. 1e, f). All data demonstrated that administration of MSCs had a tumor-suppressive effect in the Is of DEN-induced hepatocarcinoma development and a tumor-promotive effect in its Ps stage.

### Administered MSC alleviate DEN-induced liver damage and lessen compensatory proliferation in the Is of hepatocarcinoma development

**Fig. 2:**
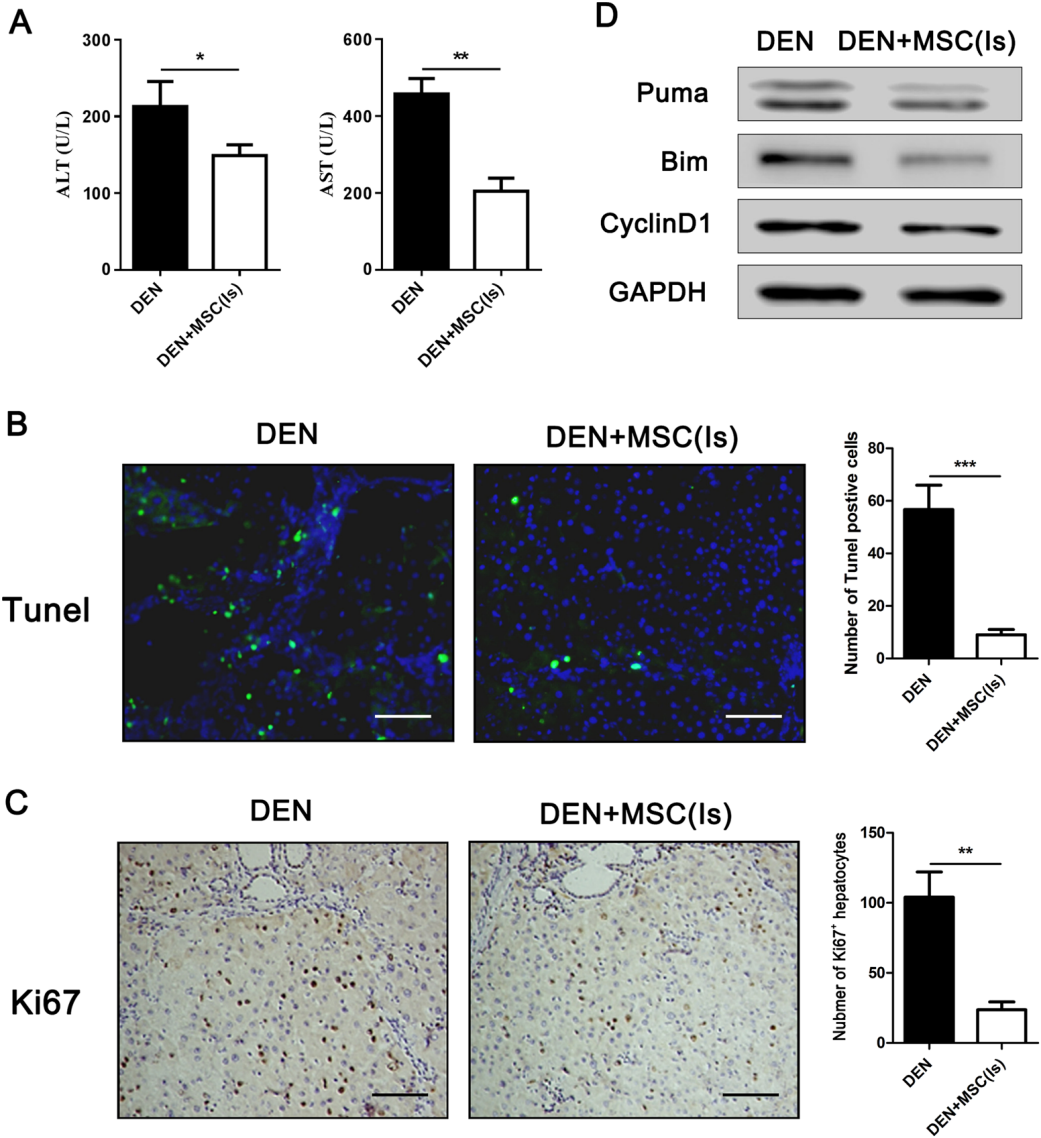
MSCs protected from liver damage and lessened compensatory proliferation in the Is of hepatocarcinoma development. **a** ALT (left) and AST (right) levels of rat serum in DEN and DEN + MSC (Is) group were tested at 8th week. **b**, **c** Tunel staining and immunostaining for Ki67 in DEN and DEN + MSCs (Is) group at 8th week. The semiquantitative analysis was shown in the graph (right). **d** At 8th week, livers of DEN and DEN + MSC (Is) were removed and lysed to assess the expression of Puma, Bim, and CyclinD1 by western blot analysis. Values are shown as mean ± SEM (*P < 0.05, **P < 0.01, ***P < 0.001). Representative of three independent experiments

Our results verified that MSCs could inhibit DEN-induced liver cancer. To further explore how MSCs affect hepatocarcinoma in the Is, serum levels of alanine aminotransferase (ALT) and aspartate aminotransferase (AST) were measured at 8th week. Much lower levels of ALT and AST were detected in DEN + MSC (Is) group, implying that MSCs could have a protective effect on liver damage in the Is of HCC development (Fig. 2a). The Is of tumor development is considered as a process of chronic tissue damage containing a cycle of cell death and death-driven compensatory proliferation^16^. We attempted to study whether MSCs affect HCC development by inhibiting cell proliferation and apoptosis. As expected, histological examination revealed that less scattered Tunel-positive hepatocyte and non-hepatocytes in the liver were detectable in DEN + MSC (Is) group than that in DEN group, suggesting that administration of MSCs in the Is may result in less DEN-induced cell apoptosis. Furthermore, liver in DEN + MSC (Is) showed a significant decrease of Ki67-positive hepatocytes compared to DEN group (Fig. 2b, c). In accordance with the above results, western blotting analysis shows that the pro-apoptotic factors Puma and Bim and proliferation-related protein CyclinD1 level were reduced in DEN + MSC (Is) group (Fig. 2d). All results indicated that administrated MSCs could protect hepatocytes from DEN damage by inhibiting cell death and death-driven compensatory proliferation.

### MSCs reduce the DNA damage and ROS accumulation of liver cell induced by DEN

**Fig. 3:**
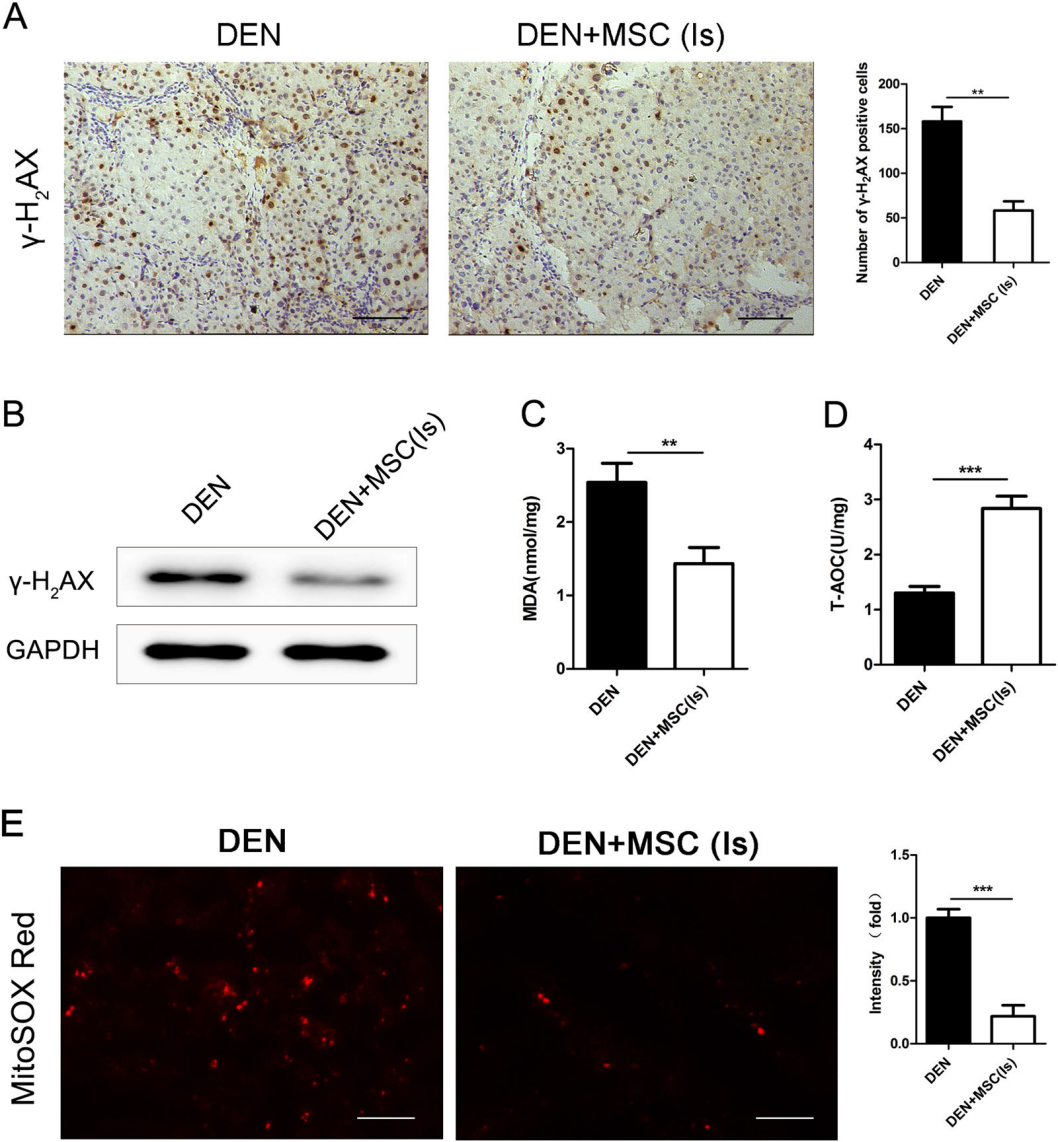
MSCs alleviated DNA damage and ROS accumulation of liver cells induced by DEN. **a** Immunostaining for γ-H_2_AX in the rat livers of DEN and DEN + MSC (Is) at 8 weeks after DEN administration. Number of positive cells was shown in the graphs (right panel). **b** Livers of rats in DEN and DEN + MSC (Is) group were removed and cell lysates were immunoblot with the indicated antibodies at 8th week. **c**, **d** Lipid peroxidation and oxidation resistance was examined at 8th week by measuring MDA **c** and T-AOC **d** in liver homogenate, respectively. **e** Liver cryosections of rats in DEN and DEN + MSC (Is) group were incubated with 2 μm MitoSOX Red mitochondrial superoxide indicator for 30 min. Staining positive cells were identified by fluorescence microscopy (left panel) and quantification is shown at right. All scale bars = 100 μm. Values are shown as mean ± SEM (**P < 0.01, ***P < 0.001). Representative of three independent experiments

Previous reports suggest that DEN administration resulted in DNA damage and ROS production in liver cells, which could contribute to cell apoptosis. Therefore, we evaluated the DNA damage and oxidative stress of liver cells at 8th week. Immunostaining of phospho-histone H_2_AX (γ-H_2_AX) showed a rapid response to DNA damage^17^. As shown in Fig. 3a, b, the number of γ-H_2_AX positive cells was significantly decreased in DEN + MSC (Is) group as compared with DEN group, which was also verified by Immunoblot analysis. Further, rat livers of DEN + MSC (Is) group had a lower content of malondialdehyde (MDA), as well as a higher level content of total antioxidative capacity (T-AOC), compared to that of DEN group (Fig. 3c, d).

The changes in MDA and T-AOC level suggested that MSCs decreased the oxidative stress in the DEN-treated rat, which was also confirmed by the MitoSOX Red staining. As shown in Fig. 3e, less extensive fluorescence was observed in DEN + MSC (Is) group, compared with DEN group. These findings indicated that administration of MSCs attenuated liver damage possibly depending on regulation of DNA damage and oxidative stress accumulation.

### MSCs in the Is exert anti-inflammatory and anti-liver fibrosis effect

**Fig. 4:**
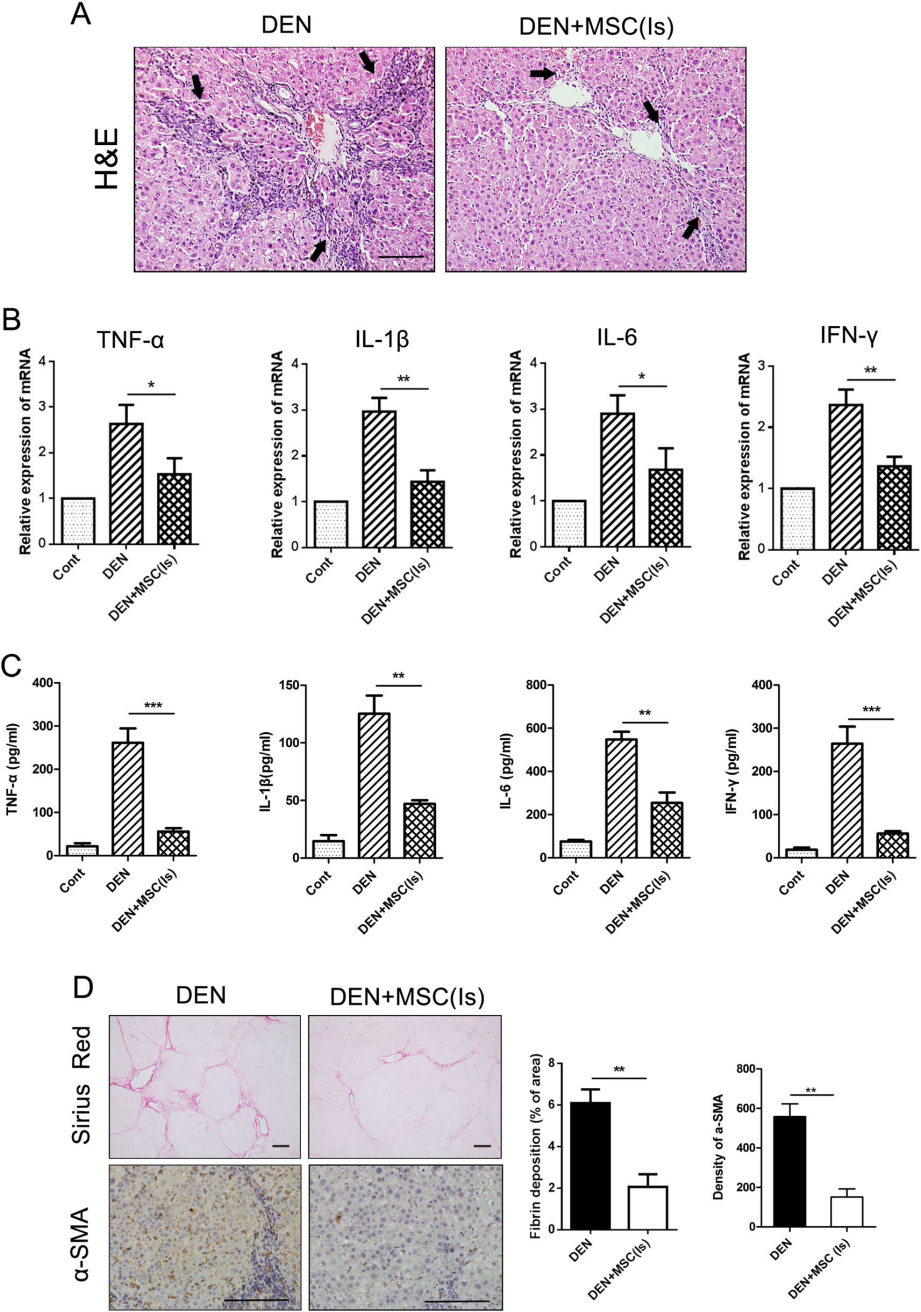
Administered MSCs in the Is exerted anti-inflammatory and anti-liver fibrosis effect. **a** Rats were fed with DEN water for 8 weeks and liver sections were stained with H&E (bar: 50 μm). **b** The expressions of TNF-α, IL-1β, IL-6, and IFN-γ mRNAs were semiquantified by real-time PCR. **c** Serum level of the cytokines was detected by the Luminex technology (Bio-Plex, Bio-Rad). **d** Sirius red staining and immunostaining for α-SMA in the rat livers of DEN and DEN + MSC (Is) group at 8th week. Quantification is shown in the graph (right). Scale bar: 100 μm. Values are shown as mean ± SEM (*P < 0.05, **P < 0.01, ***P < 0.001). Representative of three independent experiments

Inflammation was regarded as a key regulator of cancer occurrence and progression. Hence, tissue sections collected at 8th week were used to measure the inflammation in liver. We found that less lymphocytes were observed in the liver portal area in DEN + MSC (Is) group than that in DEN group (Fig. 4a). And the messenger RNA levels of inflammatory cytokines (tumor necrosis factor (TNF)-α, interleukin (IL)-1β, IL-6, and interferon (IFN)-γ) were prominently decreased in DEN + MSC (Is) group. Further, we tested inflammatory cytokine levels by microbeads based multiplex cytokine assay. We found as expected that serum levels of TNF-α, IL-1β, IL-6, and IFN-γ were dramatically reduced after MSCs administration, showing that MSCs could effectively suppressed inflammation (Fig. 4b, c).

Fibrosis is a sign of liver damage and a potential contributor to progressive cirrhosis of the liver. To further assess the extent of fibrosis, Sirius red staining was performed. The histological analysis showed that less collagen fiber accumulation and fibrotic septa was detected in DEN + MSC (Is) group. Immunostainings for α-SMA demonstrated that activated myofibroblasts were also significantly decreased in DEN + MSC (Is) group compared to that in DEN group (Fig. 4d). Thus, the tumor-suppressive effect of MSCs in the Is had a close relationship with less pronounced inflammation and reduced fibrosis.

### Administered MSCs promotes tumor cell proliferation in the Ps of hepatocarcinoma progression

**Fig. 5:**
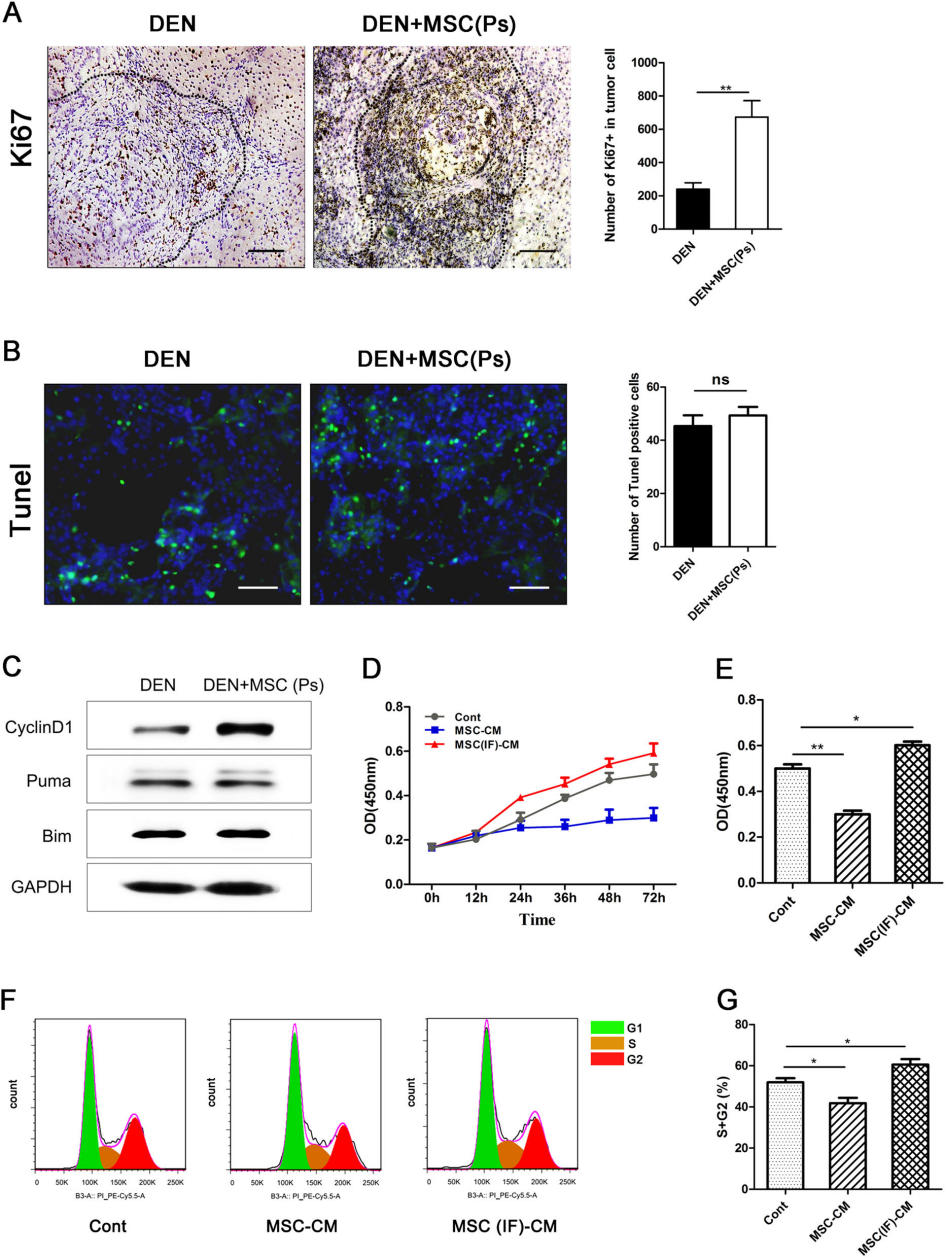
Administered MSCs promotes tumor cell proliferation in the Ps of hepatocarcinoma progression. **a**, **b** Immunostaining for Ki67 **a** and Tunel staining **b** in the liver sections in DEN and DEN + MSC (Ps) group at 14 weeks after DEN treatment. Dashed lines indicate the tumor area. The semiquantitative analysis was shown in the graph (right panel). Scale bar: 100 μm. **c** At 14th week, livers in DEN and DEN + MSC (Is) group were removed and lysed to assess the expression of CyclinD1, Puma, and Bim by immunoblot with indicated antibodies. **d**, **e** Rat CBRH-7919 cells were treated with conditioned medium of MSC (MSC-CM) or MSC pretreated by 10 ng/ml TNF-α/IFN-γ for 12 h (MSC (IF)-CM). The cell proliferation was assayed by CCk8 at different times **d** and the influence of conditional medium on 7919 cells proliferation was compared at 72 h **e**. **f**, **g** Cell cycle analysis of 7919 cells was measured by flow cytometry **e** and the fraction of S + G2 phase was shown **g**. Values are shown as mean ± SEM (*n* = 3, *P < 0.05, **P < 0.01). Representative of four independent experiments

The finding that administration of MSCs at the Ps had a tumor-promotive effect promoted us to examine the role of MSCs on liver cancer cell proliferation and apoptosis. The histological examination at 16th week revealed that the number of Ki67-positive cells increased dramatically in DEN + MSC (Ps) group compared to DEN group, suggesting that MSCs in the Ps could promote liver cancer cell proliferation. And no significant difference of Tunel-positive cells was observed between DEN and DEN + MSC (Ps) group, indicating that MSCs in the Ps failed to protect liver cells against carcinogen-induced apoptosis (Fig. 5a, b). Consistently, immunoblot analysis demonstrated that rat livers of DEN + MSC (Ps) group had a higher level of proliferation-related protein CyclinD1, and no significant difference of the expression level of Puma and Bim, compared to the DEN group (Fig. 5c).

It is yet to be determined whether MSCs were responsible for promoting cancer cell proliferation directly. The lesions in both DEN and DEN + MSC (Ps) group were consistently associated with high level of pro-inflammatory cytokines expression (Supplementary Figure 2A–D), therefore MSC-CM or MSC TNF-α/IFN-γtreated (IF)-CM were used for CCK8 and cell cycle analysis in vitro. Interesting, MSC (IF)-CM weakly hastened the growth of CBRH-7919 cells compared to control group (Fig. 5d, e). And the flow cytometry analysis showed that the fraction of S + G2 phase in MSC (IF)-CM group was more than that in control group, indicating that MSC (IF)-CM could promote proliferation of CBRH-7919 cells (Fig. 5f, g). Therefore, these results suggested that MSCs in tumor or inflammation environment was associated with the enhanced proliferation of cancer cell.

### Administered MSCs at the Ps enhance cancer stem cell properties of liver cancer cell

**Fig. 6:**
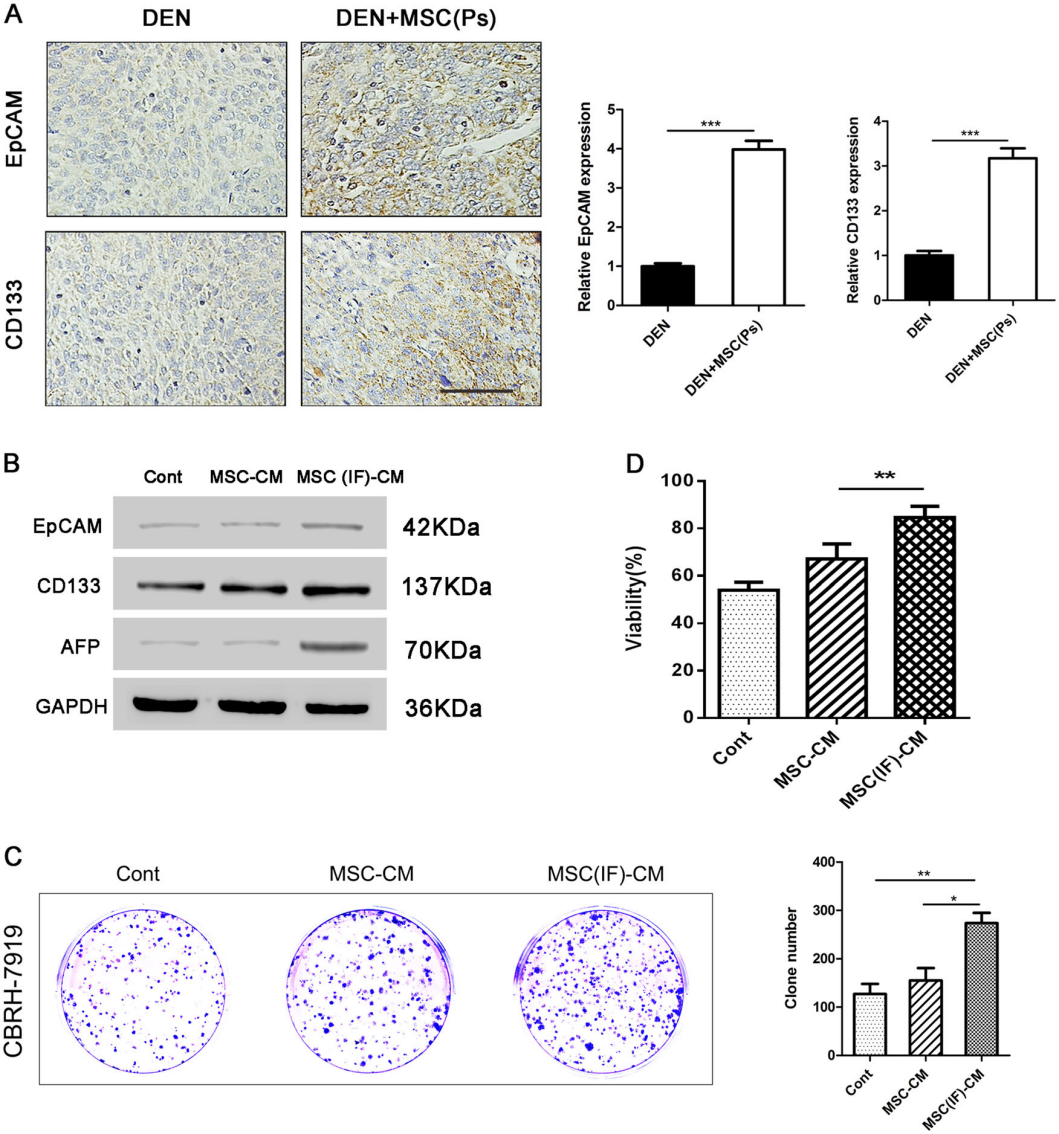
MSCs enhanced cancer stem cell property of liver cancer cell in the Ps of hepatocarcinoma development. **a** Immunostaining for EpCAM and CD133 in the rat liver of DEN and DEN + MSC (Ps) group. The positive staining was quantified by Image-Pro Plus software (right panel) (bar: 100 μm). **b** CBRH-7919 cells were treated by MSC-CM or MSC (IF)-CM for 48 h. And cell lysed to assess the expression of stem cell markers by immunoblot with indicated antibodies. **c** Clonogenic ability of CBRH-7919 cells upon the MSC or MSC (IF)-CM exposure was examined using clonogenic assay. A representative photograph is provided in the left panel, and quantified data are shown in the right panel. **d** Cell viability was measured using CCK8 assay. 7919 cells were treated with cisplatin with MSC-CM or MSC (IF)-CM exposure. Values are shown as mean ± SEM (*P < 0.05, **P < 0.01, ***P < 0.001). Representative of four independent experiments

Increasing evidences demonstrate that cancer stem cells (CSCs) are responsible for sustaining tumor growth. Therefore, the expression of CSC markers was examined in our study. As expected, a higher expression level of EpCAM and CD133 was detected in DEN + MSC (Ps) group, compared with DEN group, indicating that MSCs facilitated the stem cell property of liver cancer cell (Fig. 6a).

Next, to further verify the regulation of MSCs in stem cell property of liver cancer cell, we examined the stem cell markers and colony formation ability of CBRH-7919 cells in vitro. We demonstrated that 7919 cells treated with MSC (IF)-CM conveyed higher expression of CD133, EpCAM and AFP (Fig. 6b). Colony formation ability is the gold standard for measuring the stem cell property. After 10 days culture, CBRH-7919 cells treated with MSC (IF)-CM showed a stronger colony forming ability than the other groups (Fig. 6c). Besides, the assay for cell viability showed that only CBRH-7919 cells with MSC (IF)-CM exposure displayed stronger cell viability in response to *cis*platin compared to CBRH-7919 alone, indicating that MSC (IF)-CM could enhance the capacity of resistance to chemotherapy (Fig. 6d). All data confirmed the role of MSCs exposure to inflammation environment in contributing to stemness enhancement of liver cancer cells.

### MSCs promote EMT of liver cancer cells in the Ps of hepatocarcinoma progression

**Fig. 7:**
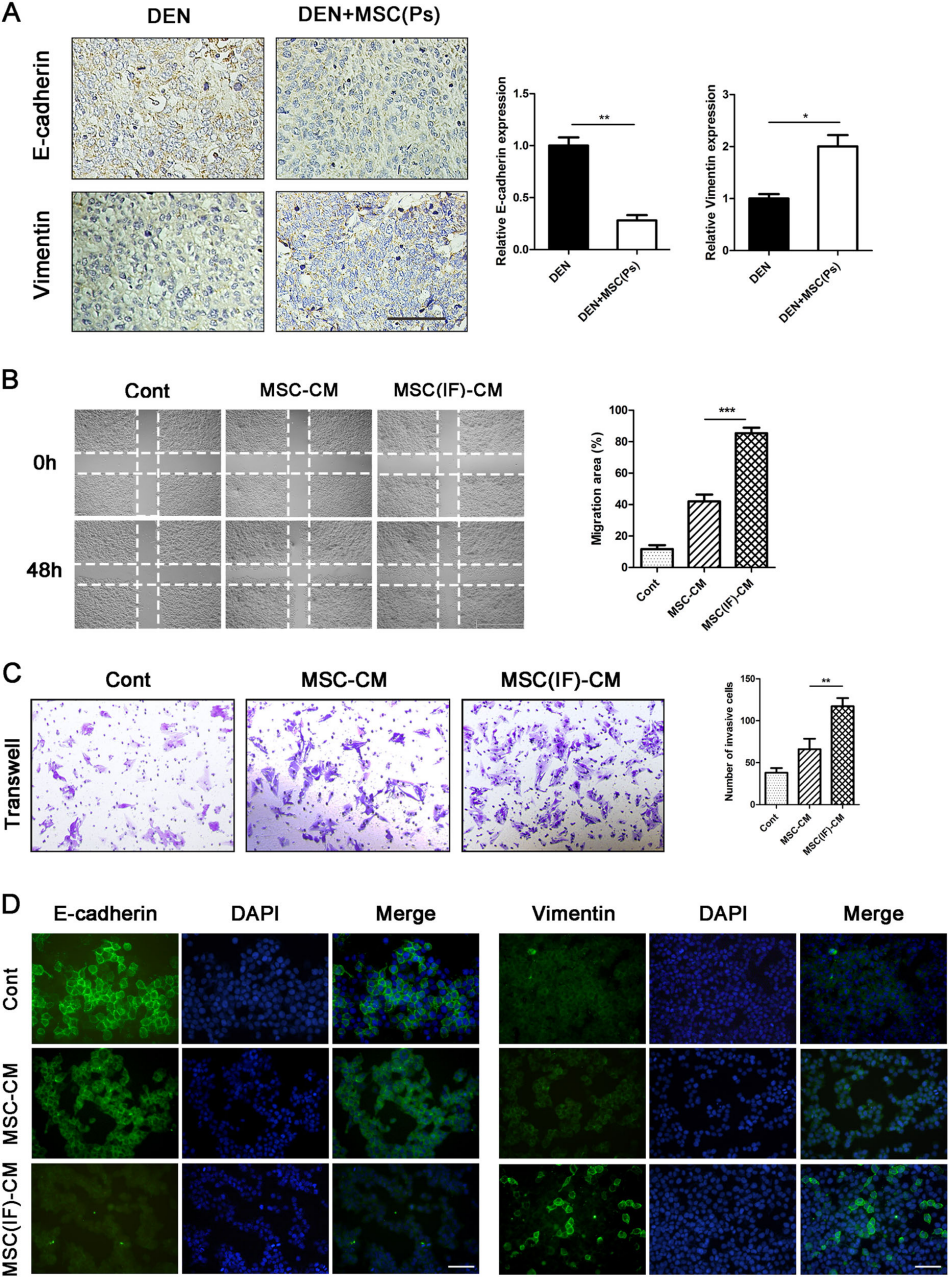
Administered MSCs promoted EMT of liver cancer cell in the Ps of hepatocarcinoma development. **a** Immunostaining for E-cadherin and Vimentin in the rat livers of DEN and DEN + MSC (Ps) group. The positive staining was quantified by Image-Pro Plus software (right panel) (bar: 100 μm). **b** The wound-healing assay was employed to determine the migration of CBRH-7919 cells exposure with DMEM medium (Cont), MSC-CM or MSC (IF)-CM for 2 days and percentage of migration area was quantified as shown in right panel. **c** Invasiveness of cells was determined using the Transwell assay. CBRH-7919 cells were plated in the upper chamber without FBS for 24 h, and MSC-CM or MSC (IF)-CM with 10% FBS was placed in the lower chamber. The number of cells that invaded through the Matrigel was counted in 10 fields under the ×20 objective lens. **d** Immunofluorescence staining was used to detect the expression of E-cadherin and Vimentin in CBRH-7919 cells (bar: 100 μm). Values are shown as mean ± SEM (*P < 0.05, **P < 0.01, ***P < 0.001). Representative of four independent experiments

Emerging evidences associate cell stemness with acquisition of epithelial–mesenchymal transition (EMT) in cancer. We found a profound reduction of E-cadherin expression and upregulation of Vimentin in DEN + MSC (Ps) group compared with DEN group (Fig. 7a). We further investigated the influence of MSCs on liver cancer cell behavior in vitro. In wound-healing assay, CBRH-7919 cell exposure in MSC (IF)-CM migrated at a much longer distance than that in MSC-CM (Fig. 7b). Next, a Transwell invasion assay was used to evaluate their invasive ability. After 24 h, CBRH-7919 cells with MSC (IF)-CM showed higher invasive ability (Fig. 7c). Further, in CBRH-7919 cells with MSC (IF)-CM, E-cadherin was remarkably decreased; while, Vimentin was robustly induced by immunofluorescence assay (Fig. 7d). Collectively, all data suggested that MSCs played a role in promoting EMT of liver cancer cell in the Ps of hepatocarcinoma development.

## Discussion

The tumor microenvironment is critical to both the initiation and maintenance of tumorigenesis^18^. Mesenchymal stem cells, as a kind of components of tumor microenvironment, can exhibit a marked tropism towards site of tumors and contribute to various tumor initiation, progressions, and metastasis, including HCC. However, there is a lively debate in the literature whether MSCs exert a pro- or anticancer action. Several studies reported that tumor progression and metastasis could be increased by MSCs^5,8,13^. In contrast, other studies showed that MSCs suppress growth of tumors, depending on the particular cancer type and animal model applied^12,19,20^. In this study, we chose the rat hepatocarcinoma model induced by DEN, which provided a multistage hepatocarcinoma development, but also simulated human primary HCC. Besides tumor heterogeneity, multiple step involved in the process of tumor development maybe the key factor to explain the contradictory role of MSCs in HCC. Therefore, in this study, we attempt to explore the effect of MSCs in different stage of HCC development using the DEN-induced rat liver cancer model.

Differing from other studies, our results showed that MSCs had paradoxical role in the different stage of HCC. In the Is of hepatocarcinogenesis, administered MSCs showed a tumor-suppressive effect, while a tumor-promotive effect in the Ps. The most likely explanation was that the microenvironment and object of transplanted MSCs was quite different between the Is and Ps. When tissue was injured, MSCs could actively integrate into damaged tissues and participate in tissue repair. We found administrated MSCs could protect hepatocyte from DEN-induced damage and finally had a tumor-suppressive effect in the Is. Importantly, at this moment, hepatocyte was the main object of transplanted MSCs. There is evidence that oxidative DNA damage, which produced by DEN in hepatocyte, is an obligatory component of carcinogenesis^21,22^. Interestingly, we demonstrated that DNA damage was significant decreased after MSCs transfusion in the Is. As such, MSCs reduced ROS accumulation and showed enhanced antioxidant capacity. Consistent with this hypothesis, Luiz and colleagues recently demonstrated that canine mesenchymal stem cell (cMSCs) overcame Thioacetamide (TAA)-induced oxidative stress in vitro, as shown by increased viability and lower cytotoxicity and ROS levels^23^. These data indicated that MSCs alleviated DEN-induced liver damage through its resistance to DNA damage and oxidative stress.

In addition, MSCs are addressed to have immunomodulatory capacity and its immunosuppressive activities are induced by inflammatory cytokines^24^. MSCs can secrete various immunomodulators to modulate the function of immune cells or directly suppress immune cell activation^25,26^. As expected, in the Is of hepatocarcinogenesis, we found that MSCs reduced the expression of pro-inflammatory cytokines and suppressed the inflammation reaction, which had a close association with hepatocarcinoma development. In fact, it is reported that ROS are produced by phagocytes in response to microbial and inflammatory stimuli^27^. On the other hand, ROS acts as signal transducing molecules that provoke the upregulation of inflammatory cytokine^28–30^. Our results showed that MSCs in the Is had a crucial role in ROS accumulation and inflammation regulation. However, whether MSCs act on ROS directly or indirectly remains unclear.

Actually, MSCs do not always promote healing, and their properties can change according to the pathophysiological status of the tissue they resided^31,32^. The interactions between MSCs and surrounding microenvironment lead to different outcomes depending on the type of tissue damage, and strength of local inflammation. As such, Yulyana et al.^12^ reported that human normal fetal MSC could inhibit HCC proliferation by sequestering free insulin-like growth factors. While, Mandel et al.^6^ demonstrated that tumor-associated MSCs (TA-MSCs) significantly promote the tumor cell proliferation. Our in vivo findings were in agreement with Mandel et al.^6^ that much more Ki67-positive tumor cells were observed in DEN + MSC (Ps). We speculate that in the Ps, the transplanted MSCs may turned to TA-MSC or had some properties of TA-MSCs induced by microenvironment, which led it act quite different from untransplanted MSCs. Interestingly, our in vitro study demonstrated that MSC (IF)-CM, mimicked TA-MSC in their microenvironment, could weakly promote the tumor cell proliferation. However, MSC (IF)-CM failed to exhibit a strong stimulus as expected, which leads us to hypothesis that the promotion effects of MSCs on HCC seemed to not only rely on improved proliferation, but also in other ways, such as regulating cancer cell stemness.

It is important to point out that CSC is closely related to cancer initiation, development, and metastasis, and its plasticity is tightly regulated by tumor microenvironment^33,34^. And EMT is addressed to be associated with the acquisition and maintenance of stem cell-like characteristics and is sufficient to endow normal cancer cells with stem cell properties. Furthermore, CSCs often exhibit EMT properties^35^. Therefore, for verified the above hypothesis, we examined the key markers of CSC and EMT in the liver samples. We found that MSCs in tumor microenvironment could enhance stemness properties of liver cancer cell by IHC staining. This notion was supported by in vitro experiments, which showed that MSC (IF)-CM treatment could strengthen colony formation ability and chemotherapy resistance of CBRH-7919 cells. However, the effect of MSC (IF)-CM in enhancing the stemness of liver cancer cell may due to increase the self-renewal ability of the original stem cell population or influence the transition of non-stem cells into stem cell, which remains to be further confirmed. In addition, our result demonstrated that MSCs in tumor microenvironment could trigger EMT in liver cancer cells, similar to the previous studies, which suggested that molecules such as hepatocyte growth factor (HGF), epidermal growth factor (EGF), and transforming growth factor (TGF)-β, secreted by MSCs, could activate a series of EMT-promoting transcription factors to transmit EMT-promoting signals^36,37^.

Many clinical applications of MSCs are proposed, either as therapeutic agents in their own property or as anticancer drug or gene vehicles^38–40^. MSCs therapy has been investigated for treating liver fibrosis. Due to its tropism for tumor microenvironment, MSCs are hoped to serve as cellular delivery vehicles for anti-tumor agents. Despite the short-term safety reported by clinical trials for liver disease, however, HCC is more complicated with several different development stage. Therefore, to elucidate the role of MSCs in HCC initiation and development is crucial for its application to treat HCC. Our study verified administration of MSCs in the Is had an anti-tumor effect, indicating that it is effective to treat chronic liver disease with MSCs. More important, our finding advised caution in the application of MSCs for HCC treatment, in that we demonstrated that administration of MSCs in the Ps could promote liver cancer growth. Therefore, the timing of MSCs introduction into the tumor microenvironment maybe critical for clinical cancer therapy. And more robust studies demonstrating the mechanisms of tumor support or suppression by MSCs may increase the utilization of MSCs in regenerative medicine without risk of promoting tumor growth and with minimal side effects.

## Materials and methods

### Animals

Male Sprague-Dawley (SD) rats weighing 180 ± 20 g were obtained from Shanghai Experimental Center, Shanghai, and maintained under pathogen-free conditions. Rats were maintained in the Second Military Medical University Animal care Committee. All animal protocols used were approved by the Institutional Animal Care and Use Committee of the Institute of Health Sciences.

### Preparation of MSCs

Rat MSCs were generated from the tibia and femur bone marrow aspirates from male SD rats weighting 200–250 g. Cells were cultured and characterized basing on our previous study^41,42^. Cells were cultured in DMEM medium supplemented with 10% FBS, 1% penicillin–streptomycin (DMEM complete medium; all from Gibco, Grand Island, NY, USA) in tissue culture flasks. Non-adherent cells were removed after 72 h and adherent cells were maintained with medium replenishment every 3 days. MSCs-GFP were obtained from GFP transgenic rats by the same method.

### Rat hepatocarcinoma model and treatment

In order to induce hepatocarcinoma, rats were given 0.1% DEN (Sigma-Aldrich, St. Louis, MO, USA) in their drinking water, along with a standard solid diet. The rats of DEN + MSC (Is) group received MSCs/MSCs-GFP (1.5 × 10^6^ per rat) at 4, 6, and 8 weeks after DEN treatment, and the rats of DEN + MSC (Ps) group received MSCs transfusion at 10, 12, and 14 weeks, respectively. Fourteen weeks later, the DEN water was replaced by normal sterile water. Some rats were killed at 14 or 16 weeks and the others were kept to be observed for survival times. Liver tumors were measured with vernier caliper and counted. Tumor volumes were calculated by the following formula: volume = *a* × *b*^2^/2 (“*a*” means the diameter at the widest point of the tumor and “*b*” means the minimal diameter).

To further assess the effect of MSCs on hepatocarcinoma, the blood was collected and serum was separated for biochemical analysis. And liver section was dissected and divided into three parts. One part was snap-frozen in liquid nitrogen for protein preservation, and the other part was soaked in TRIzol reagent (Invitrogen, Carlsbad, CA, USA) for RNA extraction. The last part was fixed in 10% formalin for histological examination.

### Immunohistochemistry and Tunel staining

For histological examination, 5 μm sections were cut and stained with hematoxylin-eosin and Sirius red according to the manufacturer’s protocol. The primary immunohistochemistry antibodies including Ki67 (1:500, Abcam), γ-H_2_AX (1:400, CST), α-SMA (1:500, Themo Fisher), CD133 (1:100, Abcam), EpCAM (1:100, Abcam), E-cadherin (1:200, Abcam), Vimentin (1:250, Abcam), GFP (1:200, Abcam) were used. For IF, after incubated with indicated primary antibody, cells were appropriate Alexa Fluor 488 or 569-labeled secondary antibodies (Invitrogen). Nuclei was counterstained with 4,6-diamidino-2-phenylindole (DAPI, Beyotime technology, Jiangsu, China). The cells were subsequently scanned with a confocal microscope (Leica TCS SP2, Germany).

Tunel staining (Calbiochem, La Jolla, CA, USA) was used to assess the apoptosis level according to the manufacturer’s instructions. Then the sections were stained with DAPI as counterstaining and observed under a fluorescence microscope.

### Western blotting

Equal amounts (10–30 μg) of cell lysate were separated by SDS-PAGE (Bio-Rad, Hercules, CA, USA), and the proteins were transferred onto polyvinyl difluorid membranes (Merck Millipore, Darmstadt, Germany). After overnight incubation with primary antibody, the membrane was washed, stained with horseradish peroxidase-conjugated IgG secondary antibody, and visualized using enhanced chemiluminescence detection reagents (GE Healthcare, USA). Hybridization with anti-GAPDH (Bioworld technology, USA) was used to confirm equal protein loading.

### Real-time PCR

Total RNA was extracted using Trizol reagent (Invitrogen) according to the manufacturer’s instruction. The 2 μg RNA input for complementary DNA (cDNA) synthesis was determined by spectrophotometric OD260 measurement and cDNA was produced using cDNA Reverse Transcription Kit (Invitrogen) as described in the instruction manual. The primer was purchased from Invitrogen as shown in Table 1. Real-time quantitative PCR reactions were performed in a 96-well optical reaction plate using a 7500 Fast Real-Time System (Applied Biosystems, CA, USA).

**Table 1:** The sequences of PCR primers

Gene		Sequence (5′–3′)
IL-1β	F	AGCTTCAGGAAGGCAGTGTC
	R	TCAGACAGCACGAGGCATTT
TNF-α	F	ATCCGAGATGTGGAACTGGC
	R	CGATCACCCCGAAGTTCAGT
IL-6	F	CACTTCACAAGTCGGAGGCT
	R	TCTGACAGTGCATCATCGCT
IFN-γ	F	GGAGATGAGCTAGAATAGAGG
	R	CATAGGAGAGGACACAGTTAT
GAPDH	F	CCGTGTTCCTACCCCCAATG
	R	CCTTTAGTGGGCCCTCGGC

### Biochemical analysis and detection of cytokines

Serum ALT and AST levels were determined using a Fuji DRICHEM 55500V (Fuji Medical System, Tokyo, Japan) according to the manufacturer’s instructions. The levels of MDA, T-AOC (all from Nanjing jiancheng Bioengineering Institute, Nanjing, China) were measured using the assay kits.

Cytokine levels in serum at 8th week were assayed by multiplexed bead array immunoassay using Luminex Technology according to the manufacturer’s protocol (Bio-Plex, Bio-Rad Laboratories, Hercules, CA, USA).

### Cell viability assay

MSC conditioned medium (MSC-CM) were collected from MSCs cultured in two-dimensional culture dishes and centrifuged at 1000×*g* for 5 min to obtain the supernatant. MSCs pretreated with IFN-γ and TNF-α was performed to mimic the inflammation environment in tumor. And MSCs were incubated with 10 ng/ml recombinant rat TNF-α/IFN-γ (Peprotech EC Ltd., London, England) for 12 h to obtain the special medium (MSC (IF)-CM). To evaluate the effect of MSC on the cell viability of rat liver cancer cell line CBRH-7919, 7919 cells (5 × 10^3^ cells per well) were seeded into a 96-well culture plate and then treated with MSC-CM or MSC (IF)-CM and exposure to *cis*platin (10 µg/ml) for 48 h. The cytotoxicity of *cis*platin was determined by cell counting Kit-8 (CCK-8, Bioworld) assay according to the manufacturer’s instructions.

### Colony formation and wound-healing assay

About 1 × 10^3^ CBRH-7919 cells per well were seeded into a six-well culture dish. After incubated at 37 °C for 2 weeks, the cells were stained with 0.1% crystal violet solution. The number of colonies containing P50 cells was counted under a microscope.

The method for wound healing has been described^43^. About 5 × 10^4^ CBRH-7919 cells were seeded in 24-well plates and incubated for 24 h, then the monolayer cells were disrupted by scratching with a 10 μl microsterile pipette tips. Photographs were taken at 0 and 48 h in a phase-contrast microscope.

### Transwell invasion assay

About 5 × 10^4^ CBRH-7919 cells in 200 μl of serum-free medium were seeded in the upper chamber. And 500 μl of MSC-CM or MSC (IF)-CM supplemented with 10% FBS were added to the lower compartment. After incubated for 48 h, cells migrated to the lower surface of the membrane were fixed with 4% paraformaldehyde, stained with 0.1% crystal violet, and counted under a microscope.

### Statistical analysis

All date are shown as mean ± SEM. Significance was assessed by unpaired two-tailed Student’s *t* test or analysis of variance. *P* < 0.05 was considered statistically significant.

## Electronic supplementary material

Supplementary Figure
