# Supplementary material for: The distinct roles of mesenchymal stem cells in the initial and progressive stage of hepatocarcinoma
Source: Cell Death Dis. 2018 Mar 1;9(3):345. doi: 10.1038/s41419-018-0366-7 (PMC5832809; doi:10.1038/s41419-018-0366-7)
Supplement: Supplementary file 1 — Supplementary Figure [file 41419_2018_366_MOESM1_ESM.docx]

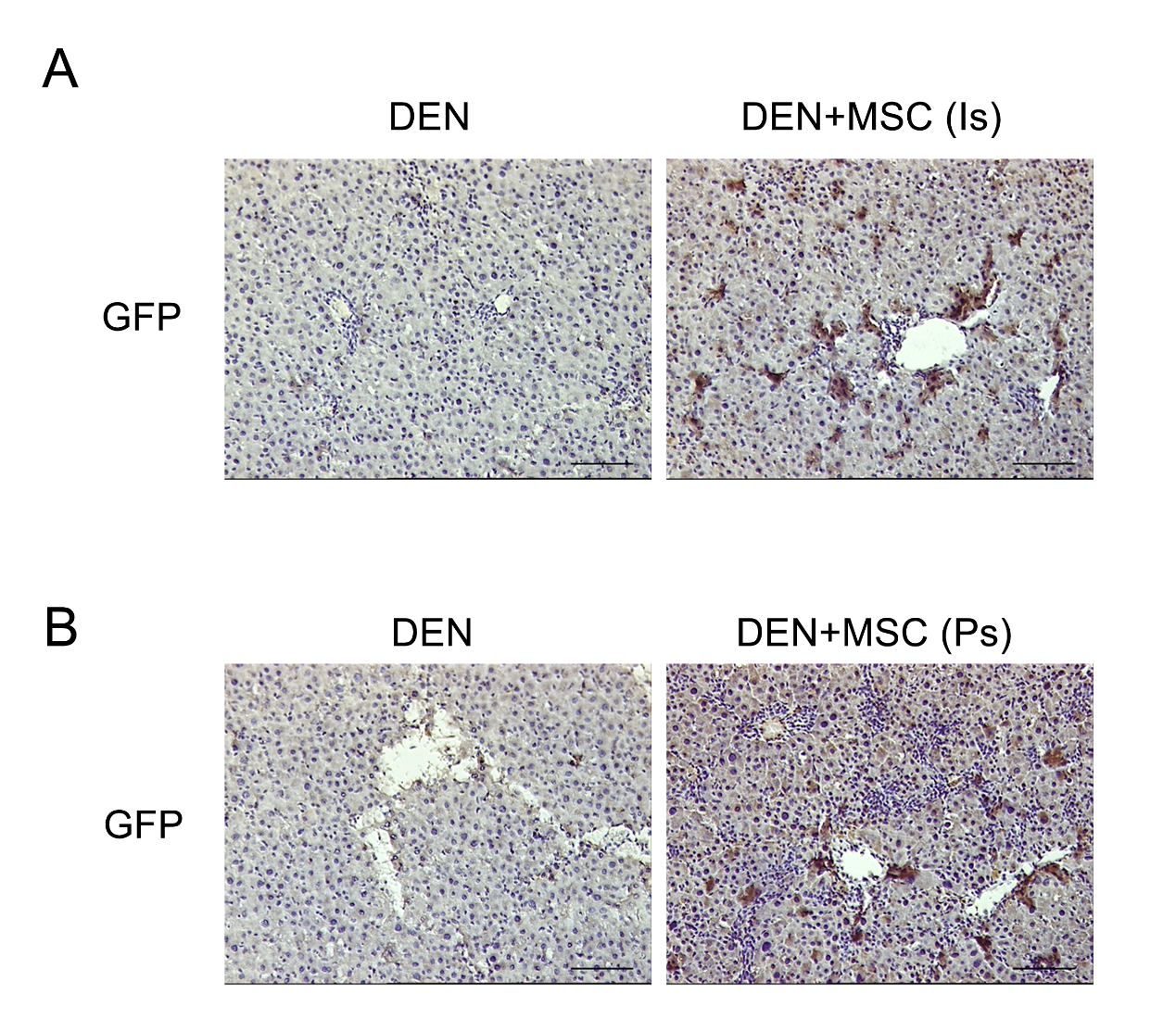


**Supplementary Figure 1**

**Tracing of administrated MSCs in the Is and Ps of hepatocarcinoma.** (A) 8 weeks after DEN treatement, immunostaining of GFP in the rat livers of DEN and DEN+MSC (Is) groups. (B) Immunostaining of GFP in the rat livers of DEN and DEN+MSC (Ps) groups at 14 weeks after DEN treatment. The GFP positive cells represent administrated MSCs. Scale bar: 100μm.


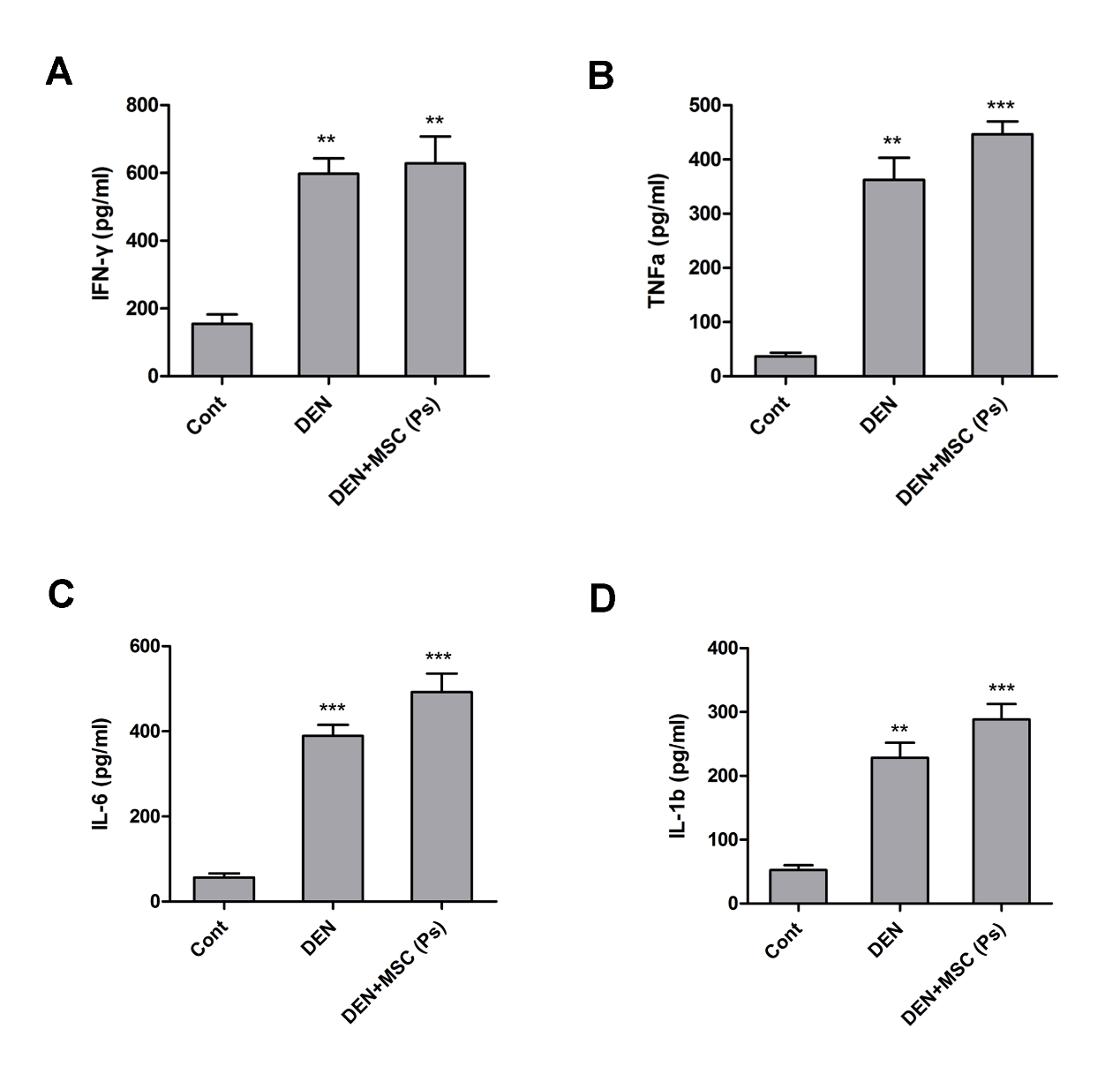


**Supplementary Figure 2**

**Rats with DEN treatment were consistently associated with high production of proinflammatory cytokines in the Ps of hepatocarcinoma development. (A-D)** Serum levels of IFN-γ, TNF-α, IL-6 and IL-1β were assayed by the Luminex technology (Bio-Plex, Bio-Rad). For this figure, values are shown as mean ± SEM, and statistical significant indicated as **P<0.01, ***P<0.001. Cont group represent rats without DEN treatment.
